# Supplementary material for: Structural and Functional Peculiarities of α-Crystallin
Source: Biology (Basel). 2020 Apr 23;9(4):85. doi: 10.3390/biology9040085 (PMC7235859; doi:10.3390/biology9040085)
Supplement: Supplementary file 1 [file biology-09-00085-s001.pdf]

Structural and functional peculiarities of α-crystallin

Supplementary Material

Olga M. Selivanova[1], Oxana V. Galzitskaya[1]*

[1] Institute of Protein Research, Russian Academy of Sciences, 142290 Pushchino, Moscow Region, Russia

*Correspondence and requests for materials should be addressed to O.V.G. (email: ogalzit@vega.protres.ru)

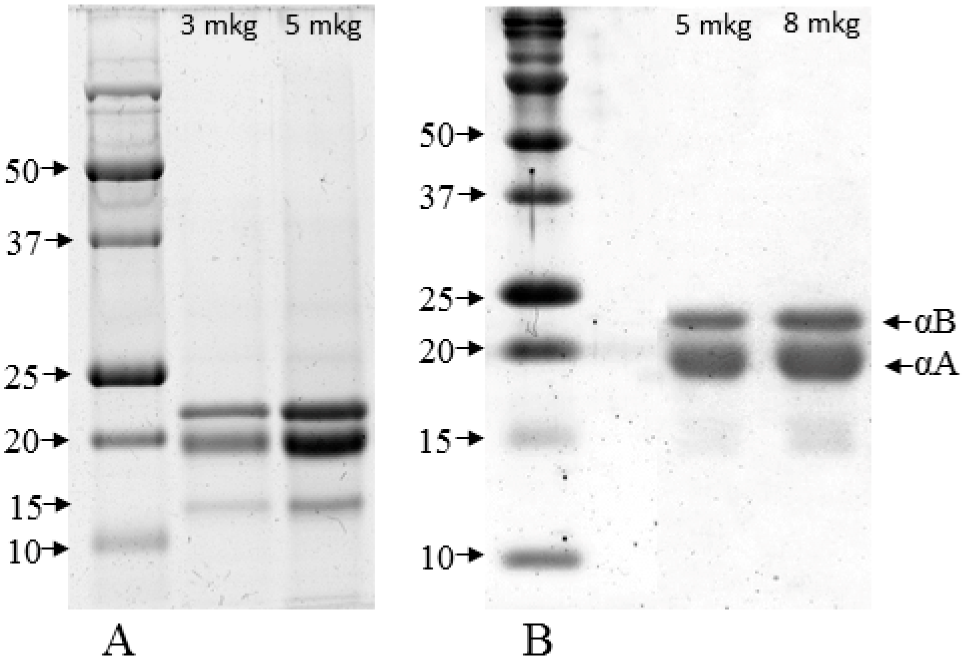

Figure S1. SDS gel electrophoresis of native α-crystallin from bovine lens (A) and from Sigma (B). 15% SDS-PAGE in tris-tricine system. Numbers, in kDa, indicate the position of marker proteins. 3, 5, and 8 micrograms (mkg) are load.

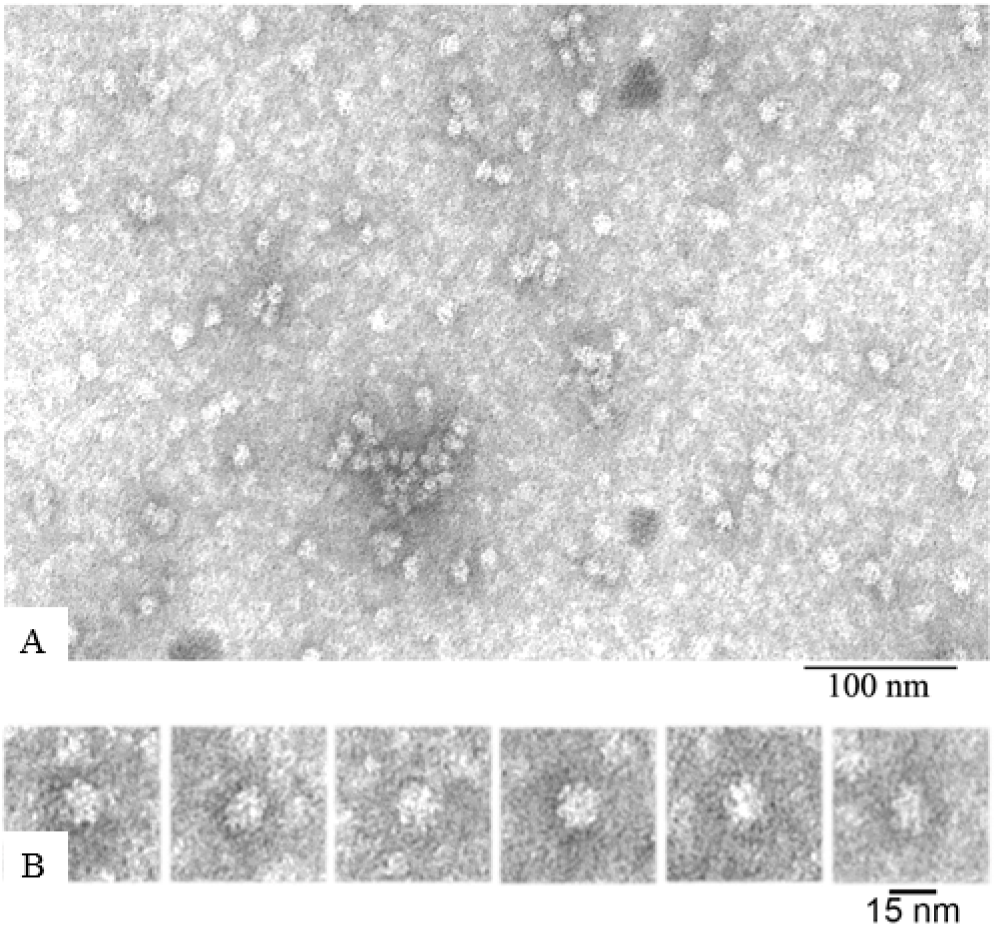

Figure S2. Electron microscopy of negatively stained recombinant αB-crystallin from bovine eye lens (sample provided by K.O. Muranov). (A) Preparation field; (B) gallery of individual αB-crystallin complexes. Preparation conditions for EM analysis: C = 0.2 mg/ml, incubation during 30 min at 37° C in 20 mM Tris-HCl buffer (pH 7.5), 100 mM NaCl, 1 mM EDTA.

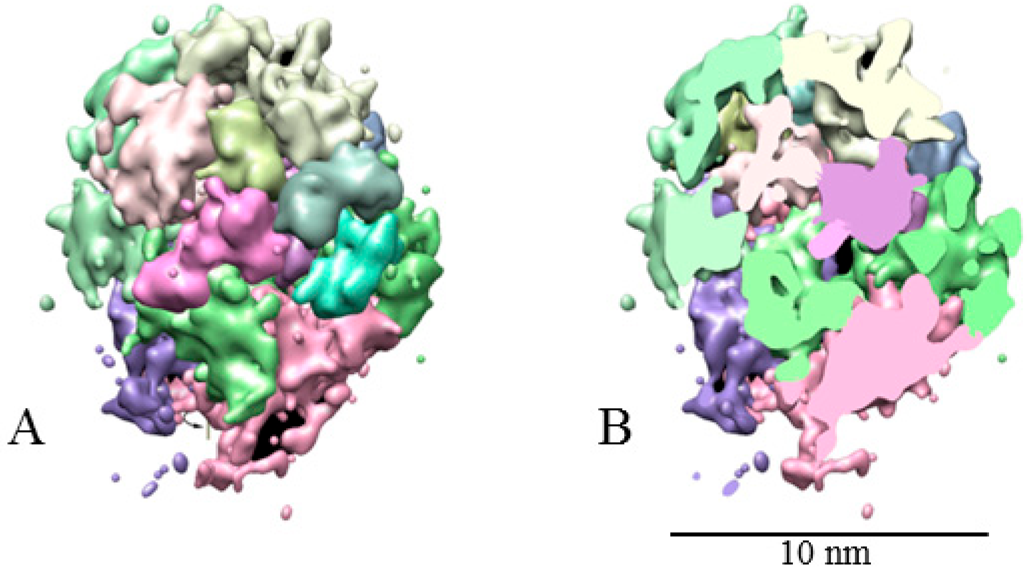

Figure S3. 3D reconstruction of negatively stained native α-crystallin complex from bovine eye lens was performed using EMAN 1.9 (Electron Micrograph Analysis) software. The resolution of the model is ~2 nm at FSC=0.5 by the EO-test (EMAN) criterion. The model was visualized in Chimera. The model was filtered with Gaussian filter at 4.2 to reduce the noise. A, Overview of the model with the molecular mass of the complex of about 650 kDa; B, Section of the complex (the middle). As seen, the model particle of the α-crystallin complex has no distinct cavity inside. When the molecular mass of the complex increases similar particles are obtained but with smaller cavities inside the particles.

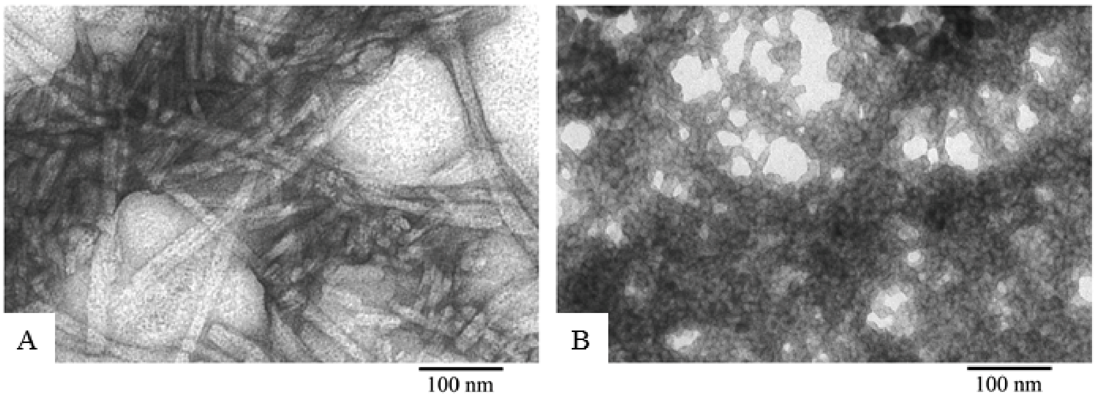

Figure S4. EM analysis of negatively stained gel preparations of the amyloidogenic AspNB fragment of the Bgl2 protein from the yeast cell wall (Biochim. Biophys. Acta 2016, 1864, 1489–1499) and a-crystallin: (A) amyloidogenic fragment of Bgl2 (5% acetic acid, pH 2)); (B) α-crystallin (20 mM Tris-HCl (pH 7.5), 100 mM NaCl, 1 mM EDTA).
